# Optimizing patient partnership in primary care improvement: A qualitative study

**DOI:** 10.1097/HMR.0000000000000250

**Published:** 2019-05-23

**Authors:** Shehnaz Alidina, Peter F. Martelli, Sara J. Singer, Emma-Louise Aveling

**Affiliations:** **Shehnaz Alidina, SD, MPH,** is Senior Global Health Systems Researcher, Department of Global Health and Social Medicine, Harvard Medical School, Boston, Massachusetts.; **Peter F. Martelli, PhD, MSPH,** is Associate Professor, Sawyer Business School, Suffolk University, Boston, Massachusetts.; **Sara J. Singer, PhD, MBA,** is Professor, Stanford School of Medicine, Stanford University, California.; **Emma-Louise Aveling, PhD,** is Research Scientist, Department of Health Policy and Management, Harvard T. H. Chan School of Public Health, Boston, Massachusetts, and The Healthcare Improvement Studies Institute, University of Cambridge, United Kingdom. E-mail: eaveling@hsph.harvard.edu.

**Keywords:** collaborative, partnership, patient-centered care, patient-centered medical home, patient engagement, practice improvement, primary care transformation

## Abstract

**Background:**

The need to expand and better engage patients in primary care improvement persists.

**Purpose:**

Recognizing a continuum of forms of engagement, this study focused on identifying lessons for optimizing patient partnerships, wherein engagement is characterized by shared decision-making and practice improvement codesign.

**Methodology:**

Twenty-three semistructured interviews with providers and patients involved in improvement efforts in seven U.S. primary care practices in the Academic Innovations Collaborative (AIC). The AIC aimed to implement primary care improvement, emphasizing patient engagement in the process. Data were analyzed thematically.

**Results:**

Sites varied in their achievement of patient partnerships, encountering material, technical, and sociocultural obstacles. Time was a challenge for all sites, as was engaging a diversity of patients. Technical training on improvement processes and shared learning “on the job” were important. External, organizational, and individual-level resources helped overcome sociocultural challenges: The AIC drove provider buy-in, a team-based improvement approach helped shift relationships from providers and recipients toward teammates, and individual qualities and behaviors that flattened hierarchies and strengthened interpersonal relationships further enhanced “teamness.” A key factor influencing progress toward transformative partnerships was a strong *shared learning journey*, characterized by frequent interactions, proximity to improvement decision-making, and learning together from the “lived experience” of practice improvement. Teams came to value not only patients’ knowledge but also changes wrought by working collaboratively over time.

**Conclusion:**

Establishing practice improvement partnerships remains challenging, but partnering with patients on improvement journeys offers distinctive gains for high-quality patient-centered care.

**Practice Implications:**

Engaging diverse patient partners requires significant disruption to organizational norms and routines, and the trend toward team-based primary care offers a fertile context for patient partnerships. Material, technical, and sociocultural resources should be evaluated not only for whether they overcome specific challenges but also for how they enhance the shared learning journey.

## Abstract 2

Supplemental digital content is available in the text.

Health systems face the challenge of improving health care quality and outcomes at the lowest possible cost. Patient engagement is increasingly promoted as one potentially cost-effective and patient-centered strategy for advancing care (Schoen et al., 2009). It is considered a foundational element of a learning health system according to the U.S. Institute of Medicine (2001), now National Academies of Medicine, and public and patient involvement is a policy requirement of the United Kingdom’s National Health Service (Department of Health, 2008). Patient engagement is a core principle and qualification standard of the patient-centered medical home (PCMH) model for delivering primary care (Scholle, Torda, Piekes, Han, & Genervo, 2010).

Patients and their caregivers possess distinctive knowledge about health and health care through their “lived experience” (Bate & Robert, 2006; Renedo, Komporozos-Athanasiou, & Marston, 2017). Support for patient engagement in health care improvement stems from both a *normative* commitment to patients’ rights to involvement in decisions about health care services and a belief in the *functional* value of patient engagement as an effective means of improving quality, efficiency, and patient-centeredness of services. However, how patient engagement is defined and operationalized is highly variable. One helpful distinction is between patient engagement in direct, individual patient care and patient engagement in practice (re)design and quality improvement (Carman et al., 2013; Scholle et al., 2010). While encouraging results from patient engagement in direct care are growing, effective involvement of patients in health care improvement processes remains limited, as does research to inform and optimize these efforts (Cené et al., 2016; Gillam & Newbould, 2016; Han, Scholle, Morton, Bechtel, & Kessler, 2013; Herrin et al., 2016; Willard-Grace, Sharma, Parker, & Potter, 2016), particularly from the patient perspective (Bombard et al., 2018). This qualitative study aimed to identify lessons for optimizing patient engagement in primary care practice improvement, drawing on provider and patient experiences.

## Conceptual Framework

Primary care practices use a variety of methods to obtain patient and family input into improvement efforts, including surveys, suggestion boxes, and patient advisors (Han et al., 2013; Sharma et al., 2016). These differing forms of engagement, ranging from consultation to shared leadership of improvement, can be conceptualized as a continuum characterizing the (re)distribution of authority and decision-making power among providers and patients (Aveling & Martin, 2013; Carman et al., 2013). Although patient consultation (e.g., through surveys) offers some advantages (e.g., minimal time burden on patients), evidence suggests this form of engagement is less likely to result in improvements to care processes, service delivery, governance, or cultural changes (Bombard et al., 2018). Consultative processes, wherein decision-making power remains in the hands of providers with little feedback or accountability to patients, also risk tokenism, which serves to legitimize predetermined plans and priorities (Bombard et al., 2018; Ocloo & Matthews, 2016). To meaningfully involve patients in leadership and decision-making for quality improvement requires sustained participation such as through membership in a patient advisory council or improvement committee (Bombard et al., 2018). Often termed *partnership* in this more transformative form of engagement, providers cede some authority and decision-making power to patients working together through dialogue to “codesign” service improvement (Aveling & Martin, 2013; Donetto, Tsianakas, & Robert, 2014). In this way, patient partnerships furnish the potential for patients’ lived experience to influence all stages of primary care improvement from problem identification to testing and implementation of solutions (Bate & Robert, 2006).

Though some encouraging results are emerging, engaging patients as *partners* in improvement is challenging (Davis et al., 2016; Donetto et al., 2014). In practice, involved patients’ ability to influence decision-making is often limited (Ocloo & Matthews, 2016), and evidence suggests that few practices—including PCMH practices—engage patients as partners in implementing primary care improvement (Han et al., 2013). One obstacle may be a perceived lack of time and other material resources (Sharma et al., 2016). Research also highlights as potential barriers technical issues such as knowledge about health systems, clinical knowledge, and familiarity with technical language, practices, and processes for managing quality improvement (Renedo, Marston, Spyridonidis, & Barlow, 2015). Training programs may address these technical challenges. However, as in any kind of improvement work, addressing technical and material challenges alone is insufficient (Bosk, Dixon-Woods, Goeschel, & Pronovost, 2009). Norms, beliefs, feelings, values, and organizational culture also influence the ways in which people engage with improvement efforts. Sociocultural challenges are particularly pertinent to patient engagement given the institutional norms, structures, and power dynamics that constrain the spaces into which patients are invited (Renedo et al., 2015). Partnering with patients disrupts traditional authority gradients and the familiar patient–provider roles established through clinical encounters. Patients may be skeptical or lack confidence, whereas providers sometimes question the legitimacy and status of patients’ knowledge (Martin, 2008). For practice improvement to be codesigned, patients and providers must renegotiate identities and norms of interaction to support dialogue, information sharing, and redistribution of decision-making power. Disrupting and remaking institutionalized norms and relational dynamics in this way requires resources to help navigate the sociocultural challenges of partnering, in addition to material and technical resources.

This study used qualitative methods to explore the experiences of primary care providers and patients engaged in practice improvement to achieve primary care goals, including high-functioning teams and patient-centered care. We focused on understanding how to optimize partnerships with patients and on identifying material, technical, and sociocultural resources that facilitated them.

## Methods

### Setting and Sample

We conducted in-depth interviews to explore patient and provider experiences of patient engagement in primary care improvement at seven primary care practices participating in the Academic Innovations Collaborative (AIC). Established in 2012, the AIC was a collaboration of the Harvard Medical School and seven academic medical centers (AMCs) that introduced change concepts central to the PCMH and an effort to establish team-based care (Chien et al., 2018). Over 4 years of the AIC, interdisciplinary providers and patients participated on 19 primary care practice-based “transformation teams” and endeavored to (a) transition to interprofessional team-based care delivery, (b) proactively manage patient populations, (c) manage care of complex patients, and (d) promote patient engagement in their care and improvement efforts. Practices received financial support and technical assistance from transformation coaches and participated in triannual collaborative learning sessions and regular webinars (Bitton et al., 2014). In the second 2 years, practices aimed to reduce missed and delayed diagnoses of breast and colorectal cancer and to improve care integration for patients with complex needs.

For this study of patient engagement in practice improvement, we selected one practice from each AMC using two criteria. The first criterion was transformation coaches’ subjective assessment of the extent to which the practices engaged patients in transformation work. Coaches had extensive experience in quality improvement methods and patient engagement and worked closely with practices throughout their transformation efforts. They were thus positioned to make an assessment because they understood both what the teams were trying to accomplish and were most familiar with the progress of all the teams. The second criterion was each practice’s 2015 PCMH Assessment score on the item that assessed the extent to which practices “obtain feedback from patients/family about their health care experience and use this information for quality improvement.” The PCMH Assessment is a widely used, 35-item self-assessment addressing change concepts for practice transformation (Poznyak, Peikes, Wakar, Brown, & Reid, 2017). To facilitate identification of effective patient engagement strategies and to exclude practices without a patient partner, we selected the practice within each AMC that performed best on these criteria. The three hospital-based and four community-based practice sites were diverse in terms of size, patients, and payment sources (Table 1).

**Table 1:** Characteristics of practices participating in this study, from 2013 (*N* = 7)

Characteristics	Overall average	Site A	Site B	Site C	Site D	Site E	Site F	Site G
Practice size								
Patient visits per year	43,390	96,294	41,010	4,191	19,000	77,000	56,113	30,000
Total staff	129	326	187	27	35	202	94	108
Primary care physician full-time equivalent, excluding residents	11.2	28.2	9.9	0.8	5.2	21.1	13.1	4.4
Panel size per primary care physician full-time equivalent	1,338	1,169	1,127	N/A	1,180	1,057	2,201	1,226
Patient race/ethnicity (%)								
White	48%	51%	10%	25%	85%	82%	44%	42%
Hispanic or Latino	16%	5%	40%	44%	1%	4%	7%	12%
Black	20%	19%	50%	18%	4%	7%	31%	20%
Asian/Pacific Islander	4%	6%	0%	2%	8%	5%	6%	7%
Other	10%	19%	0%	11%	2%	3%	12%	10%
Insurance coverage type (%)								
Medicare	13%	1%	18%	13%	14%	14%	7%	26%
Medicaid	30%	63%	4%	54%	53%	56%	5%	6%
Other coverage (private/ commercial, self-pay, other)	57%	36%	78%	33%	33%	30%	88%	68%
Practice site location								
Hospital vs. community based	N/A	Hospital	Hospital	Community	Community	Hospital	Community	Community

We purposively sampled three interviewees at each site: (a) the AIC day-to-day leader, that is, the transformation team member with the most extensive knowledge about the practice’s improvement and patient engagement efforts; (b) a patient who had been involved in the practice’s improvement work; and (c) a frontline provider team member who could provide an additional, nonleadership perspective on patient engagement efforts.

### Data Collection

Semistructured telephone interviews with 23 providers and patients from seven practices were conducted by S. A. from April to August 2016 (see Table 2). Interviews with patients explored patients’ motivation for involvement; their experience with being involved; how the practice supported them to participate; their perceived impact; and the challenges, facilitators, and lessons learned (see Supplemental Digital Content 1, http://links.lww.com/HCMR/A55, for patient interview guide). Interviews with providers explored motivations for engaging patients; attraction, selection, and preparation of patient partners; perceived impact of patient engagement; facilitators, barriers, and lessons learned (see Supplemental Digital Content 2, http://links.lww.com/HCMR/A56, for provider interview guide). Interviews lasted approximately 1 hour, were audio-recorded, and were transcribed verbatim. Research procedures were approved by the Harvard T. H. Chan School of Public Health Committee on the Use of Human Subjects.

**Table 2:** Distribution of interviewees by role

Primary position at primary care practice	*n*
AIC day to day leaders	
Physician leader	6
Behavioral health leader	1
Project manager	1
Transformation team members	
Nurse manager/nurse	4
Social work manager	1
Medical assistant supervisor/medical assistant	2
Primary care physician	1
Involved patients	7

*Note.* AIC = Academic Innovations Collaborative.

### Data Analysis

We used Braun and Clark’s (2006) thematic analysis method (supported by Atlas.ti) to analyze interview transcripts, combining inductive and deductive approaches. Initial coding focused on identifying (a) diversity of forms of patients’ engagement, (b) perceptions of the value and impact, and (c) challenges and facilitators of engagement. This initial phase proceeded by site, elaborating codes that captured each site’s experience from the perspective of providers and of patients. Codes capturing forms and perceived impact of engagement were then summarized descriptively by site. In the next phase (thematization), we iteratively compared coding *across* sites, aggregating/refining initial codes into themes that were evidenced in at least two sites in order to characterize common challenges and facilitators. Informed by our conceptual framework, we further revised these themes, distinguishing between overarching, organizing themes of material, technical, and sociocultural challenges and resources for engagement. Finally, we explored coding within *and* across sites, triangulating descriptive characterization of within-site patient engagement experiences with identified challenges and resources, from provider and patient perspectives. This facilitated further interpretation of factors influencing variation in progress toward strong partnerships.

## Results

We first describe the variation in patient engagement reported across the seven sites: perceptions of the overall experience, the range of forms of engagement, and the extent of variation in the strength of patient partnerships. Next, we describe material, technical, and sociocultural challenges to engaging patients as partners that were common across sites and resources that helped move sites toward partnership. Finally, we describe characteristics of the “shared learning journey” that emerged as central to establishing strong partnerships. Throughout, we use the term *site* (as distinct from “practice”) to encompass both providers and patients.

### Variation in Patient Engagement

Across sites, patients contributed to improvement projects focused on patient experience (e.g., booking appointments, check-in process, signage, and other aspects of the physical space), patient care (e.g., communication with patients around cancer screening), and shaping practice policies and processes (e.g., around medical marijuana or empaneling patients to primary care teams). Individual sites varied in their progress toward establishing transformative partnerships, but there was a common arc to sites’ experiences. Initially, most providers described trepidation about involving patients and concerns about which were the “right” patients to engage. Having overcome providers’ hesitation, providers and patients often experienced a difficult process of learning what roles patients could play, how, and what support they needed. Sites described trying out different ways of involving patients and an “organic” learning process. Despite frustrations along the way, recognition of the need to further improve patient engagement practices—and likely reflecting our sampling of sites—ultimately all participants in our study reported finding the experience valuable.

I think that having advisors is a geologic level advance, a seismic shift in thinking, but I think we need a lot of other components. A lot more invitational sort of dialogue. I think that this is just a beginning. (Site A Provider)

It was encouraging because we all are being heard. […] It’s also exceeding my expectations a little bit, because more is happening than I thought would happen. (Site B Patient)

Nonetheless, we found variation in the extent to which patients were engaged as partners who felt fully integrated as members of the improvement teams with shared responsibilities and decision-making power. Forms of engagement spanned the spectrum from consultation to partnership. Practices often worked with multiple patients through multiple means of engagement, with most practices combining ad hoc methods with more sustained involvement by one or more patient advisor (e.g., having one patient partner participate in improvement team meetings, with other patients participating in ad hoc focus groups or regular, practice-wide patient surveys).

Consultative forms of engagement, whereby sites solicited patient input on certain issues, were common: Over half the sites reported using consultative methods such as practice surveys, mystery shoppers, or focus groups. In all sites, patients we interviewed sat on a patient and family advisory committee (PFAC; three sites), and/or an improvement (or “transformation”) committee (five sites). Membership of these committees did not necessarily equate to partnership; at times in all sites (especially early in the process), this participation took a consultative form, such as being invited to give feedback on plans or review patient materials. Participants in three sites did describe progressing to reciprocal, collaborative engagement indicating stronger partnerships that moved sites toward codesign. Here, patients were directly involved in decision-making (e.g., survey design, improvement targets), improvement processes (e.g., Plan, Do, Study, Act [PDSA] cycles, leading focus groups with patients), and developing solutions (e.g., new processes and materials to improve cancer screening). These activities were mirrored by shared perceptions among providers and patients that patients were equal, fully integrated members of the improvement team.

They’re with us every single meeting, and they are allowed to bring items onto the agenda. So they come to every quality improvement team meeting. […] We ask them to come to learning sessions with us, to help lead focus groups with us, and we’ve asked them to even come and give talks. […] They’re an equal participant in the team like everybody else. (Site D Provider).

I mean, we’re small—see? “*We*,” that’s really a good example: I consider myself part of the “We” of [Practice Name], and I’m just a patient partner, you know. (Site D Patient)

Respondents at all sites reported frustration, setbacks, and breakthroughs and a feeling that they still had a way to travel on the journey toward real partnership. As such, sites resist neat categorization into “success” or “failure” to establish partnerships. To illustrate the extent of variation across sites, we describe two contrasting sites: one site that established a strong partnership, from the perspective of both patient and providers, and one site where patient and provider accounts indicate engagement that remained limited to more consultative forms and a weak sense of partnership. (Given the detail provided, we do not label the sites here.)

*Strong partnership*: This site had a PFAC, which met several times a year and was cochaired by a provider and a patient. The site found trying to engage patients in improvement work through this parallel structure was limiting and subsequently invited the patient partners to be part of the improvement committee; one patient became the cochair. Early on, patients were invited to provide feedback on products such as questionnaires and brochures. Over time, patients became increasingly involved in decision-making (e.g., identifying aspects of clinical processes where improvements could be made) and in implementing improvement efforts (e.g., participating in PDSA cycles). One patient partner described experiencing a change from providers wanting to carefully manage where patient partners could—and could not—give input, to a sense that providers did not want to proceed *unless* they had patients’ input. Ultimately, providers felt the value of patient engagement went beyond changes to project aims or results due to patients’ input, to positive changes in the improvement processes themselves (e.g., perceptions the improvement committee became more “collaborative”), and that patients’ involvement enhanced the team’s ability to “gain traction”—both within and beyond the practice—for their improvement efforts).

*Weaker partnership*: This site invited patient partners to be part of the site’s improvement team. Providers expressed concerns about discussing weaknesses in front of patients and felt the “real discussions” occurred outside meetings. Over time, the site transitioned to patients participating on an PFAC instead. Multiple patients were “on the books” of the PFAC, but attendance was irregular. Patients and providers characterized the patient advisor role as providing input on relevant issues through the PFAC; examples of changes that resulted largely focused on communication with patients (e.g., changes to information provided in the waiting room, patient-facing materials). Providers reported finding patient input valuable, though noted continued struggles with how to deal with input from patients that did not align with organizational priorities. Although the patient partner’s frustration—and realization—was that “things don’t happen overnight,” they nonetheless felt their input was valued and that it was positive that providers now actively seek patient input.

To better understand this variation, next we describe the lessons learned from across sites about material, technical, and sociocultural barriers and facilitators.

### Material Challenges and Resources: Finding the Time

A significant material challenge—raised by both patients and providers—was the demand on people’s time. Four sites reported attrition of patient partners, the most common reason being time commitment or timing of meetings. Improvement work involved ongoing meetings. Patients reported finding it hard to attend, given work and family commitments. Providers reported finding it difficult to strike a good balance between ensuring sufficient opportunities for two-way dialogue and patient involvement in decision-making and making unrealistic demands on patients’ time.

We started inviting the advisors to team meetings so that they would be at team meetings. Some of them were able to do that, some weren’t, over three years’ time, some of them had other commitments or this or that, so we had six instead of nine. (Site A Patient)

Improvement efforts by definition need to be iterative and happening frequently and it would be very difficult to [find] a patient partner who wanted to be here all the time [*…*] So, that’s a barrier in that they might be able to be even more helpful if they were here all the time but we couldn’t possibly ask that of someone. (Site E Provider)

Timing was particularly critical in determining which “kind” of patient could participate. Meetings during working hours excluded many patients, skewing participation toward “stay-at-home moms or older people who are retired” (Site C Provider), or those with least “economic and social requirements” (Site A Provider). “After hours” meetings offered the possibility of involving a broader demographic but created additional work on provider’s own time. Providers also noted the additional demand on their time outside meetings to support patient engagement, with the result they felt they were having to do it on their own time or on the fly.

It’s either they miss work for it or we’re at home and they would be available. The meeting times are really based around what’s convenient at the clinic. (Site G Provider)

The tension between provider needs and desires, and accommodating a more diverse population of potential partners was one that no site felt they were able to satisfactorily resolve. For providers, this appeared to fuel broader concerns about the value of patients’ input if patients were not representative. Employing multiple means of participation, as most sites did, or having a more diverse group of patients on the PFAC offered ways to diversify the range of patient perspectives, helping providers move, to some extent, beyond a preoccupation with representativeness.

In addition to having the consistent patient partners, we’ve also hosted patient focus groups on certain topics. [*…*] So patients have been engaged that way although that’s a different population based on the topic. (Site D Provider)

### Technical Challenges and Resources: Learn as You Go

Across sites, patients and providers reported initial concerns that patients’ lack of familiarity with medical terminology and clinical structures would hinder progress in the limited time available for improvement work. Although both groups described a “real learning curve” (Site E Patient), the majority also felt lack of clinical knowledge was not as great a barrier as anticipated.

Every profession has its jargon, so if I heard anything I didn’t understand, I should feel free to ask questions. And I did. And, it was a learning process. (Site D Patient)

Technical knowledge relating to quality improvement and to specific improvement projects were another obstacle to patient partnership. Providers recognized that “the training piece is huge” (Site C Provider) but felt they lacked understanding about what kind of training patients might need and how to provide it. One site drew on institutional resources, for example, hospital-wide patient advisory group processes, to train patients. Although helpful for some issues (e.g., confidentiality policy training), this did little to prepare patients for the improvement work that teams hoped to accomplish. More helpful was topic- or process-specific training that four sites offered on an ad hoc basis.

We’ll give them necessary training. So, if a patient is involved in like a colonoscopy effort, we’ll give them literature about colonoscopies and train them on process improvement [*…*] so we train them on lean and PDSAs, and that kind of thing. (Site F Provider)

AIC learning sessions provided patients and providers training on technical skills (e.g., PDSA cycles) and support to develop clarity around improvement goals and processes.

All sites characterized their experience as “organic” and “learn as you go.” Plans for orientation or technical training were rarely developed before engaging patient partners. Despite variation in the amount and nature of technical training, all patient partners emphasized how much they learned through the work itself. Although some saw this as “experiential learning at its best” (Site A Patient), it could also engender frustration, lost motivation, and misunderstandings.

### Sociocultural Challenges and Resources: Becoming a Team

Whether or not a site already had a patient advisor or PFAC, providers in all sites reported concerns and some skepticism (their own or practice colleagues’) about engaging patients as partners in improvement work. Providers described fears about disrupting clinically established norms of patient–provider interactions and felt working “with” not “for” patients countered the identity physicians’ training socialized them into. Providers in five sites expressed concerns about “airing dirty laundry”; engaging partners collaboratively would mean sharing normally hidden information.

It’s basically airing your dirty laundry kind of thing. So when the system is broken and your patients are involved, you don’t want to, yes you’re gonna fix it, but at the same time there’s the whole issue of admitting that there’s a problem with the system. (Site F Provider)

Providers in four sites mentioned fears that patients may be motivated by having “an axe to grind” (Site E Provider) or suggest changes beyond the project scope or the site’s sphere of control.

The hospital is willing to paint it white or blue but the [patient] advisors want to change the shape of the hospital, and the administration says “well that’s not in our budget, you can tell them that.” (Site A Provider)

Patients in our study suggested “single issues” were not what motivated them; regardless of how positive or negative their prior care experiences, all reported being motivated by more generalized interests in helping sites improve patient experience and patient-centeredness. Whether in foresight or hindsight, they too recognized single-issue advocacy could be problematic for partnering, because practice transformation encompassed a range of improvement targets.

If you’re going to be a good patient partner, you need to partner. And if that’s what’s the topic, then that’s where you need to go and be. If you’re not willing to do that, then probably, you don’t want to take up that role. (Site E Patient)

Patients’ initial concerns centered around uncertainty about what to expect, what was expected of them, and what their role would be. Despite much enthusiasm, most reported doubts about whether they could play a valuable role or whether providers would value their input.

I expected perhaps, and not because of anything that had transpired in my interaction on the QI team, but just because these are medical providers, they know what they’re doing; you’re just a patient—you know that kind of little attitude in my head. (Site D Patient)

Resources for addressing the challenges associated with disrupting norms, (re)negotiating roles, overcoming concerns, and securing buy-in from providers derived from external, organizational, and individual sources. External drivers, such as PCMH certification, provided some pressure for patient engagement. Providers from all sites reported that the AIC provided the activation energy for those that had not yet engaged patients in transformation efforts. Expectations set by the AIC provided a “hard edge” as sites had to account for not engaging patients, sometimes in front of peers. Softer edges of the AIC helped secure buy-in among site-level leaders and personnel: Discussions with peers provided persuasion, reassurance, and advice on navigating sociocultural challenges.

Part of it was that one of the learning sessions, it was when they started having a “bring our patient partner” like there was a big, the upcoming learning session was gonna have a big emphasis on patient partners. Then I was like, okay I just gotta do it. (Site E Provider)

Once, through our work with the AIC, we sort of learned about how other practices have done this and were given some guidance and guidelines on expectations and how to create a good experience, we were ready to jump right in. (Site D Provider)

Within organizations, leadership by committed individuals was critical for securing buy-in from other providers who were hesitant or skeptical and supporting and encouraging patients. Patient engagement “champions” sometimes acted as brokers, identifying issues where patients’ interests and providers’ readiness for change aligned.

It’s that dance to find that right place so that it matches the political commitment at [Practice], doesn’t exceed it too much because [that] can turn off people. You need an intermediary [*…*] it probably helps that it be a health care person. (Site A Provider)

I’d say that it’s really, really, really important that [site] leadership has bought into it. […] that [makes] a big difference to whether they [providers] see the value or not. (Site F Provider)

Another organizational feature that facilitated engaging patients as partners was the team-based approach to improvement. For providers, inviting patients to work on an improvement team legitimized patients’ contributions based on personal (rather than representative) qualities such as being a team player, big picture thinker, listener, and their willingness to speak up constructively. For patients too, being a good team player (e.g., confident to speak up yet open to other opinions) was an important part of their perceptions of what constituted a “good” patient partner. But more than this, “teamness” became a symbolic resource through which to legitimize their place, transforming the hierarchical, dyadic dynamic of the patient–provider relationship.

It brings up a team dynamic and frankly it’s just hard to find the right people, right? We talk a lot about that [*…*] You really want somebody who’ll be both critical but [who’s] also gonna be helping you move things forwards. (Site B Provider)

Being [on the improvement team] made me look at it like ‘I’m not just a patient right now but I’m also partner so I need to look at things differently so I can be able to provide good feedback. (Site C Patient)

Transitioning to a team-based model of care had been a core part of the AIC program, and this existing emphasis on teamness likely represented fertile ground for engaging patients in teams. The process of building interprofessional teams disrupted “old” interprofessional dynamics and spurred renegotiation of roles, norms, and relationships among personnel. Explicit efforts to counter hierarchical dynamics adopted by some practices—such as not using titles, reducing jargon—enhanced patients’ involvement as full, effective team players.

They were trying to become a team themselves, and have everybody feel comfortable with one another from all these different roles at different levels. You know, as opposed to a hierarchical system. So, right away there was an immediate acceptance, and I would say that was good preparation in and of itself. I was told right up front, “We don’t use titles, so please do not call me Doctor because we all come to the table equally. Also, ask any questions.” (Site E Patient)

Other supportive team practices included inviting patients to participate in wider practice or improvement team activities and informal socializing opportunities to help strengthen relationships.

We even do things like invite our patient partner to staff meetings periodically so they’re right there with the rest of the group. We’ve asked them to our holiday parties, we’ve really thought about how to deeply integrate them into all things family practice, so that they’re comfortable with us, we’re comfortable with them and there’s a relationship that’s built. (Site D Provider)

I went in like okay I’m just gonna give you guys feedback, I come to the meetings once in a while and not more than that. So when I became involved, we had weekly meetings that we have to attend, and then there was the conferences we got invited to. So each time, we were part of the entire process. […] I wasn’t expecting to be part of that, a team like that. (Site C Patient)

### Variable Progress: A Shared Learning Journey

**Figure 1:**
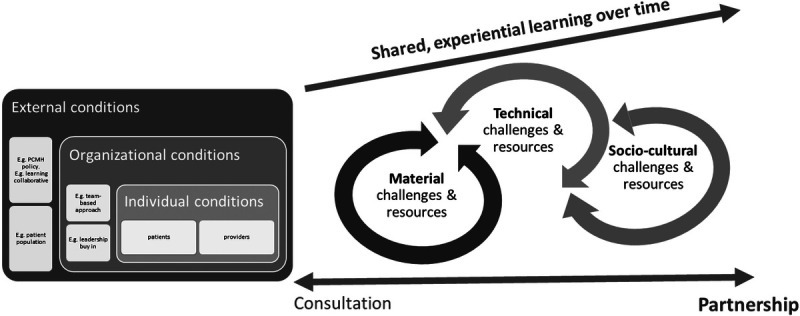
Conceptual model of the shared learning journey toward establishing partnerships with patients for practice improvement

Triangulating variation in patient engagement among sites, the challenges sites faced and resources they utilized, suggests a key factor influencing progress toward transformative partnerships and codesign was the strength of the *shared learning journey* (Figure 1). Strong journeys were characterized by three interrelated features: frequent interactions over time, proximity to improvement decision-making and activities, and learning together from the “lived experience” of pursuing practice improvement.

These features required some starting conditions. Given the time demands of regular attendance at meetings during business hours, an obvious prerequisite was patient(s) with the capacity and interest to participate. Within the practice, a minimum of political will was required (whether furnished by internal leadership and/or external drivers) to recruit patient partners and invite them into the “laundry room” of the improvement work. Alone, this was insufficient to secure strong partnerships: The sites with the strongest partnerships were three of five sites with regular, consistent involvement of patient partners in improvement team activities. What then differentiated these sites?

For patients and providers, individual “team player” qualities were important. Patients in stronger partnerships were sufficiently confident in an unfamiliar clinical setting to speak up. Among AIC practices, the preexisting quality of “teamness” varied (as described in Kyle, Aveling, & Singer, 2018). However, our data suggest these were not immutable properties that teams and individuals either possessed or lacked: Both provider buy-in and patients’ confidence in their team role evolved over time, and greater initial skepticism did not always lead to weaker partnerships.

That’s another piece that I’ve seen grow and change where, rather than concerned as to whether patients were going to compromise reaching the goals on schedule to quite the opposite: “we can’t reach our goals unless we touch base with the patients.” (Site A Patient)

Our analysis suggests the experience of working together *over time* functioned in instrumental and relational ways to strengthen partnerships. Simply working together and getting to know each other was valuable for relationship building. Over time, patients’ presence became normalized: Providers’ concerns about airing dirty laundry gave way to appreciation for patients’ participation. Patients too recognized the value of time for building relationships with providers.

We were afraid and worried about certain things, like worrying about what our patients would think of us, or recognizing that we’re not perfect, seeing some of our inner challenges. We were really concerned, and none of those fears have been founded. (Site D Provider)

When you’re with somebody all day, as opposed to one hour a month, you get to know more about that person. You get more time to find out a little bit about the person, and what they do, and how they value their work, but also personally. So it creates a bond. (Site E Patient)

Early and direct involvement in the improvement work facilitated bidirectional communication, technical learning, and mutual understanding to support shared decision-making. For patients, working alongside providers familiarized them with clinical language, structures, and local norms that enhanced participation and increased opportunities for influence. Including patients in discussions facilitated shared understanding of improvement priorities and limits. Only involving patients in parallel structures (e.g., PFACs) did little to develop such understanding, risking frustration over patients wanting changes not aligned with practice or institutional priorities and constraints.

A very important thing is to have the feedback loop, in that if you’re asking for feedback you then give feedback as to how that was incorporated or heard [*…*] there should [be] options for patients to work on solutions to problems, not just identifying the problems. (Site A Patient)

There is at least one issue [recently] where the PFAC was like ‘this is really important’ and our leadership team was like “yeah that’s not gonna happen,” and kinda feeling, we again, like weren’t sure how to navigate that one. (Site B Provider)

Beyond acquisition of technical knowledge, of central importance to the shared journey was *experiential* learning over time – for patients and providers – about the value of patient involvement and the kinds of expertise patients could provide.

It’s helping me see things in a whole other light. I propose a problem, and a solution, and I hear back we already tried that, or how do you think we should do that? It’s challenged me to come up with more solutions and think about things a little bit outside the box. [*…*] It’s been eye opening. I’m seeing a lot that I would have never seen or thought. (Site B Patient)

Deriving from the “lived experience” of engaging patients in improvement processes, this “organic process” engendered a virtuous cycle, whereby the more involvement patients had, the greater the experiential learning about the potential for and value of patient involvement and the greater the potential to reinforce relationships.

To what degree essentially is the team integrating them into the work that’s being done in the clinic? So if [“x” improvement process] is the work the team are doing right now, are the patient partners able to join that work? Is the team understanding what value they can offer? Because you have to understand the value they can offer in order to give them more access, which will pull them in as far as you possibly can. (Site G Provider)

Equally important was that patients and providers were learning *together*. Not only were patients learning about primary care practice improvement, but also providers were learning how to do this work *with* patients. The context for patient engagement was thus not straightforwardly one of provider–expert and patient–novice: Learning *together* strengthened and equalized the partnership.

They [providers] themselves were on a learning curve, and the sense was “well, we’ve got to get up to speed before we bring a patient on board.” And I was able to have a conversation with some of the people who were doing that to say, “Well, actually, we can be part of that learning piece, it’s not a question of being ready for us—we can be part- we can travel that learning curve with you, you don’t have to be prepared for us.” (Site A Patient)

We don’t prepare them. Just like us, we aren’t prepared. We go in, we have ideas and we form as a group. So it is really formed by all of us. (Site D Provider)

These characteristics were mutually reinforcing, having a catalyzing effect such that the more the patient became involved in team activities, the higher the quality of the partnership. Teams with such “catalyzed” learning journeys came to appreciate not only patients’ distinctive perspectives but also the changes to improvement processes wrought by the experience itself. These sites felt partnering led to a more collaborative style of engagement generally that benefitted communication, meeting attendance, and inclusion of traditionally lower status staff such as medical assistants, and a shift to a deeper understanding among providers of the meaning of “patient-centered care.”

[The impact on staff has been] we have other team members who otherwise wouldn’t have spoken up, they’re speaking up and giving ideas because they do understand that they’re part of a team even though they are a medical assistant, [that] their input is also valuable because they’re part of the patient experience. (Site C Provider)

We underestimate the value of someone telling you and reflecting back to you how your practice feels to the person getting that care, you know? So that’s number one [impact] and I actually think that’s probably the biggest one. [*…*] it was important in humanizing the way the decision making committee makes decisions – that’s not a small thing. [*…*] patients get more humane care because of the advisors. (Site A Provider)

It was very interesting for me over the years that I was involved specifically in the collaborative because there really was a shift and an openness—as opposed to, “we the healthcare providers have to make sure we’re doing our best by the patients”—to “we the healthcare providers have to listen to what our patients have to say.” (Site A Patient)

## Discussion

Sites varied in their achievement of patient partnerships, with only a few sites able to fully leverage patient experiences to codesign improved processes. We identified material, technical, and sociocultural challenges and resources for navigating them that derived from external, organizational, and individual sources. Many of obstacles and facilitators resonate with existing literature; for example, the value of an external collaborative in providing both hard and soft edges to galvanize initiation of patient engagement and increase buy-in among providers (Aveling, Martin, Armstrong, Banerjee, & Dixon-Woods, 2012; Schiff et al., 2016). At the organizational and individual levels, supportive practices (e.g., early, direct involvement in spaces where decision-making and implementation took place; provider behaviors that flattened hierarchies and promoted inclusive team dynamics) resonate with the wider literature on partnership and team building (Aveling & Martin, 2013; Renedo et al., 2015). In addition, individual patients’ qualities, like self-confidence in an unfamiliar professional context and willingness to engage in work for which they had little technical training, endowed them with the ability to contribute meaningfully.

Our findings extend the literature on optimizing patient partnerships in two important ways. First, reflecting wider efforts to transition to team-based care, practices in our study adopted a team-based approach to improvement. Previous studies have noted the value of technical knowledge for enabling patients to “re-organize their patient identity and master their ‘participant’ role to increase their influence” (Renedo et al., 2015, p. 30). We showed that “teamness” was a further, crucial resource that simultaneously supported patients *and* providers to renegotiate relationships, from providers and recipients toward teammates. Thus, behaviors and strategies that enhanced team functioning generally also enhanced patient participation. The experiences of these sites suggest there may be mutually reinforcing benefits of team-based care and patient engagement, because both entail disruption to established norms and engage participants in cultural change processes oriented to redistributing authority and flattening hierarchies (Kyle et al., 2018; Sheridan et al., 2018).

Organizational emphasis on teamness also offered a novel strategy for displacing persistent concerns about “representativeness” (Martin, 2008) by instead foregrounding the value of “team player” qualities. However, although functional for those involved, it did not resolve the exclusion of certain patients from partnering by virtue of, for example, the need to regularly attend meetings during working hours. As Ocloo and Matthews (2016) point out, patient partners are not often drawn from the population groups that commonly experience poorest access to quality care. Committing to addressing issues of equity and diversity in patient partnerships will likely require greater disruption, such as having meetings outside of working hours and offering financial compensation for patient partners (Sharma et al., 2016). Although difficult to ask of a workforce suffering high levels of burnout (Jha et al., 2019), it may be necessary to achieve truly patient-centered care.

Second, our study highlights the value of sustained patient involvement in a shared learning journey. Patient partnerships added value for improvement through both patients’ lived experience as patients and providers’ lived experience of patient engagement. Although sites saw value in using a variety of forms of engagement, the value of developing sustained relationships reinforces earlier work arguing that partnering with patients to codesign improvement offers distinctive benefits derived from what patients know *and* from what patients and providers learn through collaborating over time (Aveling & Martin, 2013). The shared learning journey helped expand patient and provider understandings of the value and potential impact of partnership for primary care transformation.

Given necessary, but not sufficient, conditions of patient availability, interest, and political will in practices, three mutually reinforcing characteristics of the shared journey (frequent interactions over time; proximity to improvement process decision-making and activities; and mutual, experiential learning) engendered a catalyzing, virtuous cycle. Greater involvement facilitated greater mutual understanding and experiential learning about the different kinds of contributions team members could make, motivating further, more transformative forms of engagement. The facilitative resources we identified thus derive their value not only from overcoming specific technical, material, or sociocultural challenges but also from the extent to which they enhanced a shared learning journey. For example, the most effective strategies for overcoming patients’ deficits in technical knowledge (e.g., early involvement in improvement meetings) were also significant relationally, strengthening relationships and team cohesion. Similarly, the value of practical and discursive resources furnished through learning sessions were maximized when patients also participated, promoting shared learning and social bonding. Through instrumental and relational means, the experiential learning gained through partnering was transformative in ways that supported primary care goals such as high-functioning teams and patient-centered care.

### Limitations

The sites involved in this study may be somewhat unusual in that they were involved in a 4-year learning collaborative. However, the increasing use of learning collaboratives and wider trends in the U.S. primary care context—such as the trend toward a PCMH model emphasizing team-based care, capacity for continuous improvement, and patient engagement—suggests that the findings may nonetheless be applicable in other settings. Moreover, the ubiquity of the “journey” metaphor in reports of improvement projects more broadly (Bate, Mendel, & Robert, 2008) suggests providers need not be improvement novices for there to be shared learning with patients. In practice settings that lack some of the facilitative starting conditions enjoyed by sites participating in the AIC, additional interventions, such as training or external facilitation, may be useful in tackling the sociocultural obstacles to partnership (Bombard et al., 2018).

Findings were based on experiences reported through interviews; complementary observational and longitudinal data collection would likely enhance understanding of the forms of engagement practiced and the evolving dynamics of learning journeys. Interviews with patients who had chosen to discontinue their involvement would offer another valuable perspective. A further, important aim of future research will be to investigate whether participants’ perceptions of changes in relationships and understandings of patient-centered care is reflected in improved outcomes and experiences for the wider patient population.

## Implications for Practice

Our study identified individual, organizational, and externally derived resources for tackling the material, technical, and sociocultural obstacles to partnering with patients for practice improvement. Movement toward team-based primary care represents a fertile context for patient partnerships, which may have mutually reinforcing benefits for team functioning. The significance of shared learning journeys suggests potential resources be evaluated not only for how they may overcome specific challenges but also for how they may enhance that journey. It also implies the need for an openness to nondeterministic approaches to practice transformation, accepting that understandings of “the problem” and of roles may evolve in unpredictable ways. Evolving understandings of how, when, and where patients could contribute suggest caution about well-intentioned efforts to prepare “for” patients and to match patient experiences to improvement projects or roles, which could become predetermined constraints that stifle potential benefits of patient partnerships.

Beyond the instrumental value of patients’ contributions based on their lived experience as patients, professionals’ “lived experience” of partnering with patients on improvement journeys offers distinctive gains for high-quality patient-centered care. Yet enabling participation of diverse patients in partnerships (as opposed to consulting) requires significant disruption to organizational norms and routines, and such levels of participation may not be realistic or desirable for all patients. This tension suggests the need to articulate a theory of change to determine which forms of engagement to pursue for which improvement ends.

## Supplementary Material

SUPPLEMENTARY MATERIAL
