# Supplementary material for: Optimizing patient partnership in primary care improvement: A qualitative study
Source: Health Care Manage Rev. 2019 May 23;46(2):123–34. doi: 10.1097/HMR.0000000000000250 (PMC7919701; doi:10.1097/HMR.0000000000000250)
Supplement: SUPPLEMENTARY MATERIAL [file hcm-46-123-s001.docx]

**PATIENT INVOVLEMENT IN PRACTICE TRANSFORMATION: INTERVIEW GUIDE FOR PATIENTS**

**Interview Protocol**

**Introductory Comments**

Thank you for taking the time to speak with me. My name is Shehnaz Alidina. I am a research associate at the Harvard T.H. Chan School of Public Health. The purpose of this interview is to learn more about your experiences with patient involvement in your primary care practice’s transformation efforts as part of the Academic Innovations Collaborative. The interview should take no more than 60 minutes. I’m going to take notes and record the conversation so I can review it, but I will never share anything you say in connection with your name or the name of your practice or health system. You’re welcome to skip any questions that you would rather not answer. If you don’t understand any of the questions, please let me know and I will explain them. Please remember that we want to know what you think and feel and that there are no right or wrong answers. Is it ok with you to proceed?

**Background**

1. Can you tell me how you are involved in [name of practice]’s transformation efforts?
   1. How did you hear about opportunities to become involved?
   2. How long have you been involved in improving care at this practice?
   3. What is your role?
   4. What are your responsibilities?

**Motivation**

1. Why did you get involved in efforts to improve the way care is delivered at [name of practice]? (What motivated you?)
2. What (or how) do you think patients can contribute to improving care?

**Experience with being involved**

1. Can you give me an example of a project where you felt you added value?
2. Thinking about this project:
   1. What about this experience worked well?
   2. What do you feel like you were able to contribute?
   3. What did you find frustrating or challenging?
   4. Was the experience what you expected? In what way was it or was it not?
3. Can you give me an example of a time where you felt your opinion was not heard, valued, or taken seriously?
4. What have you found hard (or disappointing) about participating in [practice’s] transformation team?
   1. Can you describe a time that felt particularly hard?

**Practice support for patient involvement**

1. Did the practice do things to help prepare you for participation in the transformation team?
   1. How well prepared for the role did you feel when you began?
   2. Does the practice do things to support your ongoing involvement?
2. What makes it difficult for you to participate in transformation team work?
   1. How well informed do you feel about transformation effort?
   2. What mechanisms does the practice use to keep you informed?
3. Given your experience, what would you like people working in primary care to know that would help them to be effective in encouraging or supporting patient involvement in transformation or quality improvement efforts?

**Closing comments**

1. What advice would you give to a patient thinking of getting involved in a transformation team at this or another health system?
2. What advice would you give to providers thinking of involving patients in their improvement efforts?
3. What is the most important message that you want us to take away from this interview?
4. Are there any other comments you would like to share that may not have come up in our conversation today?

Thank you for your time and participation in this interview. The information that you provided to us will be very helpful in this project.
