# Supplementary material for: Optimizing patient partnership in primary care improvement: A qualitative study
Source: Health Care Manage Rev. 2019 May 23;46(2):123–34. doi: 10.1097/HMR.0000000000000250 (PMC7919701; doi:10.1097/HMR.0000000000000250)
Supplement: SUPPLEMENTARY MATERIAL [file hcm-46-123-s002.docx]

**PATIENT INVOVLEMENT IN PRACTICE TRANSFORMATION: INTERVIEW GUIDE FOR PROVIDERS**

Thank you for taking the time to speak with me. My name is Shehnaz Alidina, and I am a research associate at the Harvard T.H. Chan School of Public Health. The purpose of this interview today is to learn more about your experiences with patient involvement in your primary care practice’s transformation efforts as part of the Academic Innovations Collaborative. The interview should take no more than 60 minutes. I’m going to take notes and record the conversation so I can review it, but I will never share anything you say in connection with your name or the name of your practice or health system. You’re welcome to skip any questions that you would rather not answer. If you don’t understand any of the questions, please let me know and I will explain them. Is it ok with you to proceed?

**Background**

1. What is your position at [name of practice?]
   - How long have you been with [name of practice]?
   - What are your responsibilities on your practice’s transformation team?

**Motivation**

1. What prompted your organization to engage patient partners in your practice’s transformation efforts?
   - What do you think previously stopped your practice from involving patients in similar activities?
   - What obstacles to effectively involving patients did you foresee?
   - What concerns did you have about involving them?
   - What was it that convinced you do involve patients then/when you did?
   - What were you hoping to get out of involving patients in transformation/ improvement efforts?

**Experience with involving patients in practice transformation (and practice support/learning)**

1. How did you go about involving (a) patient(s) in your transformation process?
   1. How did you identify a patient(s) (what were you looking for in the patient?)
   2. What did you invite them to do?
   3. What, if anything, did transformation team leaders do to help prepare patients to partner with the transformation team?
   4. What, if anything, did team leaders do to help prepare transformation team members to involve patient partners in the improvement work?
   5. What, if anything, have practice leaders done to promote or support patient partner involvement in practice transformation efforts?
2. Can you give an example of an improvement project at this practice that you felt was successful?
   1. In what ways, if any, did patients contribute to the success of this project?
3. Can you give an example of a time when you felt a patient’s involvement in an improvement project was particularly helpful?
   - What did the patient contribute?
   - What about the process in this example helped the patient to contribute?
4. How have your processes for transformation work changed since involving patients? (Why did you make these changes?)
   1. What methods or processes, if any, does the transformation team use to promote patient partner involvement (e.g., advance preparation)?
   2. How do you keep the patient partner(s) fully informed about the transformation effort?
5. Can you give an example of a time when involving a patient in a transformation effort felt particularly challenging?
   - What about this experience didn’t work?
   - Have any of your original concerns about involving patients in transformation efforts been borne out?
   - If you could do it over again, what would you do differently?
   - In your opinion, what are the main barriers to effectively involving patients in improvement efforts?

**Impact**

1. How would you characterize the impact of involving patients in your practice’s transformation efforts: small, medium, large, or transformational?
   - Are there opportunities you think your practice might have been missed if patients had not been involved?
   - Are there things you believe your practice is doing differently because it involved patients in practice transformation?
   - What, if at all, do you perceive the impact of involving patients in practice transformation has been on patients themselves?
   - What, if at all, do you perceive the impact of involving patients in practice transformation has been on providers and staff (e.g., change or evolution in roles, job satisfaction, workload impact)?

**Closing**

1. What are the most important lessons that you have learned about involving patients in practice transformation efforts?
2. What advice would you give to providers thinking of involving patients in their organization’s improvement efforts (to help them to be effective in encouraging or supporting patient involvement in transformation or quality improvement efforts)?
3. What advice would you give to a patient thinking of getting involved in a transformation team at this or another health system?
4. What is the most important message that you want us to take away from this interview?
5. Is there anything else that you would like to add about any of the topics that we've discussed or other areas that we didn't discuss but you think are important?

Thank you for your time and participation in this interview. The information that you provided to us will be very helpful in this project.
